# A Nanobody of PEDV S1 Protein: Screening and Expression in *Escherichia coli*

**DOI:** 10.3390/biom14091116

**Published:** 2024-09-04

**Authors:** Zhipeng Hao, Xufeng Dong, Zhongtao Zhang, Zhihua Qin

**Affiliations:** College of Veterinary Medicine, Qingdao Agricultural University, Qingdao 266109, China; hz.peng@stu.qau.edu.cn (Z.H.); dongxufeng@qau.edu.cn (X.D.); 20222113035@qau.edu.cn (Z.Z.)

**Keywords:** PEDV, nanobodies, soluble expression, *Escherichia coli*

## Abstract

Porcine epidemic diarrhea virus (PEDV) has caused significant economic losses to the pig farming industry in various countries for a long time. Currently, there are no highly effective preventive or control measures available. Research into the pathogenic mechanism of PEDV has shown that it primarily causes infection by binding the S protein to the CD13 (APN) receptor on the membrane of porcine intestinal epithelial cells. The S1 region contains three neutralization epitopes and multiple receptor-binding domains, which are closely related to viral antigenicity and ad-sorption invasion. Nanobodies are a type of single-domain antibody that have been discovered in recent years. They can be expressed on a large scale through prokaryotic expression systems, which makes them cost-effective, stable, and less immunogenic. This study used a phage display library of nanobodies against the PEDV S1 protein. After three rounds of selection and enrichment, the DNA sequence of the highly specific nanobody S1Nb1 was successfully obtained. To obtain soluble nanobody S1Nb1, its DNA sequence was inserted into the vector Pcold and a solubility-enhancing SUMO tag was added. The resulting recombinant vector, Pcold-SUMO-S1Nb1, was then transformed into *E. coli* BL21(DE3) to determine the optimal expression conditions for the nanobody. Following purification using Ni-column affinity chromatography, Western blot analysis confirmed the successful purification of S1Nb1 carrying the solubility-enhancing tag. ELISA results demonstrated a strong affinity between the S1Nb1 nanobody and PEDV S1 protein.

## 1. Introduction

Porcine Epidemic Diarrhea Virus (PEDV) is a highly contagious infectious disease characterized by vomiting, diarrhea, dehydration, and anorexia [1,2], which can occur at various stages of pig growth. Piglets affected by the disease are particularly severe, with mortality rates reaching up to 100% in piglets under two weeks of age [3,4].

Porcine Epidemic Diarrhea was first reported in the United Kingdom in 1971, followed by reports in countries such as Belgium, the Netherlands, Germany, France, Switzerland, Bulgaria, and Hungary [4]. Europe gradually controlled the disease by 2000. Currently, the disease is more prevalent in China, South Korea, and Japan [5]. China first reported the disease in 1973 [6], and successfully isolated and identified its pathogen PEDV in 1984 [7]. PEDV is a single-stranded positive-sense RNA virus, belonging to the order Nidovirales, family Coronaviridae, subfamily Coronavirinae, genus Alphacoronavirus, which belongs to the same order, family, and subfamily as SARS-CoV-2 [8]. For a long time, PED has caused significant economic losses to the pig farming industry in various countries, and there are currently no ideal preventive or treatment methods. From 2017 to 2021, in a prevalence survey in China, the positive detection rate of PEDV in pig farms remained above 50% [5]. Although vaccines have certain clinical efficacy in prevention and control, the difficulty in prevention and control remains significant, which is closely related to strain variation and the emergence of new strains [9]. The S protein is located on the surface of PEDV and is the main neutralizing antibody epitope [10]. Variations in the S protein are the main cause of antigenic variation in PEDV, which may result in reduced or even lost effectiveness of traditional vaccines [11].

The S protein is a spike protein, which forms protrusions on the viral envelope under electron microscopy, exhibiting a crown-like appearance. It belongs to the type I transmembrane glycoproteins, consisting of 1383 amino acids, with a molecular weight ranging from 180 kDa to 220 kDa. The S protein contains the major antigenic neutralization epitopes of PEDV, playing a crucial role in interacting with cellular receptors to mediate virus entry and induce neutralizing antibodies in the natural host (Bosch et al. [12]; Chang et al. [13]; Godet et al. [14]). Therefore, the S glycoprotein will be the primary target for developing effective PEDV vaccines. Although the S protein lacks protease cleavage sites, it is artificially divided into two structural domains: S1 (antigenic region, 1–735 aa) and S2 (membrane fusion region, 736–1383 aa). The S1 region is involved in receptor binding, while the latter mediates virus-cell membrane fusion [15]. The S protein contains four neutralizing epitope regions, with three of them located within the S1 region, which also concentrates multiple receptor-binding domains [16,17], closely related to viral antigenicity and adsorption invasion. Previous studies have demonstrated that monoclonal antibodies against S protein expressed in mammalian cells exhibit good neutralizing effects and can effectively inhibit PEDV through oral administration [18]. Studies on the pathogenic mechanism of PEDV have found that it primarily causes disease by binding the S protein to the CD13 (APN) receptor on the membrane of porcine intestinal epithelial cells, leading to entry into the cells. PEDV interacts with the S1-CTD domain of the S protein with APN [19]. The enzyme activity of APN promotes PEDV replication [20]. However, studies have shown that pigs with knocked-out APN genes are susceptible to PEDV infection but not to TGEV, which uses APN as a cell receptor. APN is not essential for PEDV entry into cells [21,22], so the molecular mechanism of PEDV pathogenesis still requires further investigation.

In 1993, Hamers-Casterman et al. discovered for the first time in a camel body a type of heavy-chain antibody (HCAb) that only consists of heavy chains, lacking light chains and the first constant region CH1 (conventional region of heavy chain 1). The single-domain antibodies obtained by cloning their variable regions are known as nanobodies (Nbs) [23].

In 1985, the pioneering scientist George P. Smith introduced the phage display technology [24], which involves inserting target gene fragments into the coat protein gene of bacteriophages using genetic engineering techniques. During the growth of bacteriophages, the target protein becomes a part of the outer coat protein, displayed on the surface of the bacteriophage. Through appropriate screening methods, the sequences with the strongest affinity to the target can be obtained. Currently, this is a commonly used method for screening specific nanobodies from nanobody libraries.

Nb is not naturally present in camel serum; it is only in the N-terminal variable region of heavy-chain antibodies (HCAbs) [25,26]. Nanobodies have many advantages compared to conventional antibodies: the relative molecular weight of Nb is around 12~15 kDa, only about 10% of the mass of conventional antibodies (the mass of traditional IgG is approximately 150 kDa, and HCAbs are approximately 95 kDa). It is currently the smallest antibody with complete antibody functionality [25,27,28,29]. The Nb has some important differences compared to the VH of traditional antibodies. In comparison to VH, four amino acid residues in framework region 2 (FR2) of Nb are replaced. These positions are conserved noncanonical VH structural domains [30], and these four amino acids are hydrophilic, making the surface of Nb more hydrophilic, thereby increasing its water solubility [31]. Because of Nb’s small molecular weight and high solubility, its ability to penetrate tissues is significantly enhanced compared to traditional antibodies, and it can even penetrate the blood–brain barrier. Due to these reasons, Nb has a rapid metabolism and a short half-life in the body, with most being rapidly excreted by the kidneys, and only a small fraction being metabolized in the liver [32]. Therefore, sometimes it is necessary to extend its time in the body to use Nb more efficiently. Nanobodies exhibit remarkable stability under extreme external conditions [33], it can form different conformational modes to protect the stability of amino acids. Under extreme environmental conditions, such as high temperatures or extreme acidity or alkalinity, the structure of traditional polymeric antibodies will change, involving the unfolding of two Fab arms connected to the Fc domain of the antibody through the hinge region, resulting in the loss of higher-order structure (HOS) or the disruption of secondary and tertiary structures, thus losing its original function [34]. Unlike traditional antibodies, after chemical and thermal denaturation, Nb can refold and form a disulfide bond between CDR1 and CDR3, improving the stability of its structure and ensuring the stability of its functional activity [35,36,37]. This provides good conditions for the storage and transportation of Nb. The homology of the framework region 2 (FR2) of Nb with the human VH region exceeds 80%, and its conformation is stable. Nb naturally lacks the Fc segment, eliminating the risk of bystander immune cell activation and antibody effector function, and does not induce complement reactions, making it less immunogenic and more tissue-compatible [38].

Although Nb has three CDRs like traditional antibodies, its CDR3 is longer than VH’s. The antigen-binding sites of traditional Fab fragments are concave or planar, so they can only recognize surface antigens. In contrast, Nb has a protruding antigen-binding site primarily composed of a CDR3 loop, which can bind to concave antigenic epitopes. Therefore, Nb can specifically recognize some hidden antigenic epitopes that traditional antibodies cannot recognize [39]. Compared to traditional monoclonal antibodies, nanobodies have a smaller molecular weight, simpler structure, and do not require glycosylation, making them easier to modify and produce. Bacteria and yeast cells can both serve as host cells for nanobody production. However, using yeast for production may lead to issues of excessive glycosylation. Nanobodies can be expressed in *E. coli* to yield large quantities at low cost and are easily purified [40].

Nanobodies have immense potential to serve as neutralizing antibodies. Therefore, we expressed highly specific antibody clones from the nanobody library targeting the PEDV S1 protein in *E. coli*, providing crucial materials for exploring therapeutic methods for PEDV.

## 2. Materials and Methods

### 2.1. Materials

Glucose, triethanolamine, glucose, and NaCl were all purchased from Sinopharm Group (Beijing, China). Further, 1M Tris-HCl, PBS, PEG6000, skimmed milk powder, IPTG, Dialysis Membranes (Retained molecular weight 15,000), Anti-His Tag Monoclonal antibody, Goat Anti-Mouse IgG/HRP were purchased from Solarbio (Beijing, China). Ultrafiltration centrifugal tubes (MWCO 10 kD) were from Millipore (Burlington, MA, USA). Kanamycin and ampicillin were purchased from Sangon Biotech (Shanghai, China). His-tag purification kits and BCA protein assay kits were purchased from ComWin Biotech (Beijing, China). All chemicals used were of analytical grade. Multiskan Spectrum was from Tecan (Shanghai, China).

BL21(DE3) was purchased from Sangon Biotech, TG1 cells, camel nanobody phage display library, and M13KO7 helper phage are all preserved at the Shandong Provincial Key Laboratory of Preventive Veterinary Medicine.

### 2.2. Method

#### 2.2.1. Phage Library Rescue

Inoculate single colonies of TG1 into 2 mL of 2× YT liquid medium and cultivate on a shaker at 37 °C and 200 rpm until the optical density (OD_600_) reaches 0.6. Add 100 μL of the M13KO7 helper phage at a MOI of about 20 to the TG1 liquid and incubate at 37 °C for 10 min. Transfer the culture to 100 mL of 2× YT (supplemented with 1% kanamycin) and shake overnight at 200 rpm. Centrifuge the overnight culture at 6000× *g* for 30 min at 4 °C, harvest the supernatant, and then add 1/5 volume of pre-cooled PEG/NaCl and incubate on ice for 5 h. Centrifuge again at 6000× *g* for 30 min, discard the supernatant, harvest the precipitate, resuspend in an appropriate amount of PBS, and incubate at 4 °C for 2 h. Finally, collect the supernatant by centrifugation at 10,000× *g* for 10 min to obtain the amplified phage. Dilute the phage for titration and use the upper semi-solid medium to determine the titer of the phage by observing plaque formation.

Inoculate 300 μL of phage display vector library into 100 mL of 2× YT medium (containing 1% Amp and 20% glucose) and shake at 37 °C until the OD_600_ reaches approximately 0.6. Add approximately 20 MOI of phage and allow to stand at 37 °C for 30 min. Centrifuge the mixture at 3000× *g* for 10 min, collect the bacteria, transfer to 200 mL of 2× YT/Amp-Kana medium, and shake at 37 °C and 200 rpm for 12 h. Centrifuge the culture at 4000× *g* for 30 min at 4 °C, carefully collect the supernatant to avoid aspirating the precipitate, add 1/5 volume of PEG/NaCl solution, mix well by inversion, and incubate on ice for 5 h. Then, centrifuge at 6000× *g* for 30 min at 4 °C, discard the supernatant, resuspend the phage precipitate in an appropriate volume of PBS, and incubate at 4 °C for 2 h. Finally, collect the supernatant by centrifugation at 12,000× *g* for 10 min at 4 °C. The titer of the library is calculated by colony counting on LB-Amp solid plates using the dilution plating method.

#### 2.2.2. Screening of Nanobody Library

Diluted PEDV S1 protein was added to each well of an ELISA plate, with PBS used as a blank control. The plate was incubated overnight at 4 °C to allow protein coating on the bottom of the plate. The next day, the ELISA plate was washed three times with 1× PBST and then blocked with 2.5% skim milk for 2 h. After blocking, the plate was washed four times with PBST. The phage library was diluted to 5 × 10^11^ pfu/mL and added to the ELISA plate, followed by incubation for 2 h. Subsequently, the plate was washed four times with PBST and then three times with PBS. Each well was incubated with 100 μL of triethylamine, left at room temperature for 10 min for elution, neutralized with an equal volume of 1M Tris-HCL, and the titer of the eluted solution was determined using the double-layer agar plate method. The eluted solution was mixed with TG1 cells and incubated at 37 °C for 1 h. Then, the mixture was transferred to 2× YT/Amp-GLU medium and cultured at 37 °C with shaking at 220 rpm until the OD_600_ reached approximately 0.6. The phage was amplified and concentrated for the next round of screening using the phage rescue method described above. The process was repeated for three rounds of selection, and the enrichment of each round was assessed by titer determination on indicator plates.

#### 2.2.3. Recombinant Nanobody Crude Extraction and Acquisition of Nucleotide Sequences

Mix the eluted phage from the third round with TG1 in equal volumes and spread on LB/Amp-GLU solid medium. Randomly select 48 single colonies and shake culture for 8 h. Inoculate 1% of the culture into TB medium and culture to logarithmic phase, then induce overnight with a final concentration of 1 mM IPTG. The next day, centrifuge to collect bacteria and wash once with PBS. The supernatant after bacterial cell disruption and centrifugation is the crude extraction of nanobodies.

Coat the bottom of an ELISA plate with PEDV S1 protein, and block it with 2.5% skim milk for 2 h. Incubate with crude extract for 1 h, followed by incubation with E-tag primary antibody (diluted 1:2000) for 1 h, and then with HRP-conjugated secondary antibody (diluted 1:10,000) for 1 h. Add TMB substrate for color development for 15 min, stop the reaction with 3M sulfuric acid, and measure the absorbance at 450 nm using Multiskan Spectrum. Samples with results greater than three times that of the negative control will be sent to Sangon Biotech (Shanghai) Co., Ltd., for sequencing, using Nb universal primers.

#### 2.2.4. Construction of Nanobody Recombinant Vector

**Figure 1:**
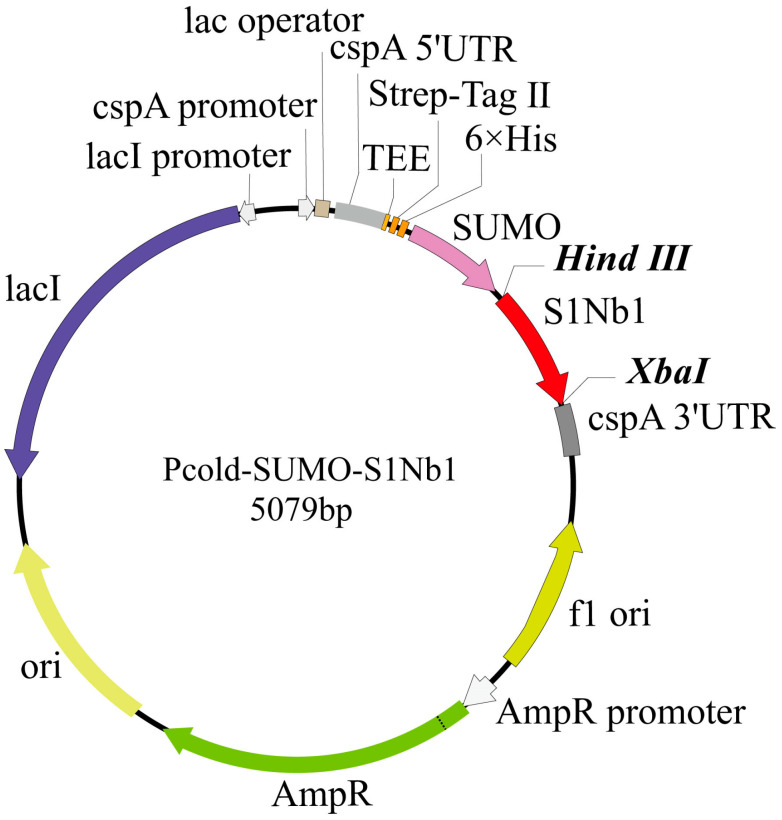
Schematic diagram of the Pcold-SUMO vector structure, expressing S1Nb1 with SUMO solubility tag, 6× His, Strep-TagⅡ tag, and TEE enhancer-promoter.

Transformation of the recombinant vector: The complete gene recombinant vector was constructed using the Pcold plasmid, with a SUMO solubility enhancement tag, a 6xHis tag, and a TEE translation enhancer added upstream of the recombinant protein to enhance its solubility expression. The entire structure was synthesized by BGI Genomics and named Pcold-SUMO-S1Nb1 (Figure 1). The recombinant expression vectors Pcold-SUMO-S1Nb1 and Pcold-SUMO were separately transformed into competent BL21(DE3) cells. Based on the recombinant vector, a pair of specific primers was designed for PCR identification, which can be used to distinguish the recombinant vector, empty vector, and untransformed cells. PCR electrophoresis was used to identify positive transformed cells. Positive clones were sequenced to ensure no nucleotide changes in the recombinant.

#### 2.2.5. Expression and Purification of Recombinant Nanobodies

To explore the optimal expression of the recombinant protein, we conducted gradient tests with different IPTG concentrations, temperatures, and induction times. The transformed positive monoclonal *E. coli* BL21(DE3) was inoculated into 5 mL LB medium (containing 1% Amp) and shaken at 37 °C for 12 h. The culture was inoculated into 500 mL LB medium containing 1% Amp at a ratio of 1% and cultured at 37 °C with shaking at 200 rpm until the optical density (OD_600_) reached 0.6. The culture was cooled in an ice–water mixture for 10 min, followed by a 20-min incubation at 15 °C to ensure uniform internal temperature. IPTG was added to a final concentration of 0.5 mM to the medium, and the culture was incubated at 15 °C with shaking at 160 rpm for 16 h. The cells were centrifuged at 6000 rpm for 15 min at 4 °C, and the pellets were washed twice with PBS before being resuspended in protein lysis buffer. The cells were sonicated on ice for 20 min at 40% power with a 3-s on and 5-s off cycle. After clarification, the supernatant containing soluble proteins was separated from the pellet by centrifugation at 12,000 rpm for 10 min and subjected to SDS-PAGE identification.

The above supernatant was purified through the Ni+ affinity resin column. To reduce non-specific binding with the purification column, the equilibration buffer was mixed with the sample in equal volumes and slowly dripped through the column to allow the His-tag to adsorb to the column. After washing with equilibration buffer, impurities were eluted with 80 mM imidazole elution buffer, followed by elution of the nanobody bound to the column with 400 mM elution buffer. The eluate was collected and dialyzed in PBS using a dialysis bag, followed by concentration using ultrafiltration. Each component was subjected to SDS-PAGE for identification and analysis. The concentration of the purified and concentrated protein was quantified using the standard Bradford protein analysis method.

#### 2.2.6. Identification of Affinity of Recombinant Nanobodies

Dilute S1 protein to 5 μg/mL with PBS, add 100 μL to each well of a 96-well ELISA plate and add only PBS to the control wells. Incubate overnight at 4 °C to allow S1 protein to coat the bottom of the plate. Wash three times with PBST, ensuring each wash is complete by tapping the liquid out of the wells onto absorbent paper. Add 2.5% skim milk to each well and incubate at 37 °C for 2 h to block the bottom of the ELISA plate. After washing with PBST, add diluted primary and secondary antibodies, 100 μL each per well, and incubate at 37 °C for 1 h each. Add TMB substrate solution for color development and incubate at 37 °C in the dark for 15 min. Add 50 μL of 3M sulfuric acid to each well to stop the color development, and measure OD450nm value.

## 3. Results

### 3.1. Phage Library Rescue

The titer of amplified phage was approximately 4.4 × 10^12^ PFU/mL, as calculated by observing the plaques on the semi-solid agar medium. The titer of the rescued recombinant library was approximately 5 × 10^11^ PFU/mL.

### 3.2. Screening of Nanobody Library

Using PEDV S1 protein as a ‘bait’ protein, we screened for nanobodies with high affinity for it. We performed three rounds of selection on the nanobody display library to increase the enrichment of nanobodies. After each round of selection, the phage titer eluted from the S1 protein was measured using serial dilution, and the recovery rate was calculated based on the input. The enrichment results are shown in (Table 1). Each round of selection increases the proportion of phages displaying a strong affinity to the S1 protein. However, to maintain phage diversity, the number of selection rounds should not be excessive. We successfully enriched high-titer nanobodies through selection.

### 3.3. Acquisition of Nanobody Nucleotide Sequences

**Figure 2:**
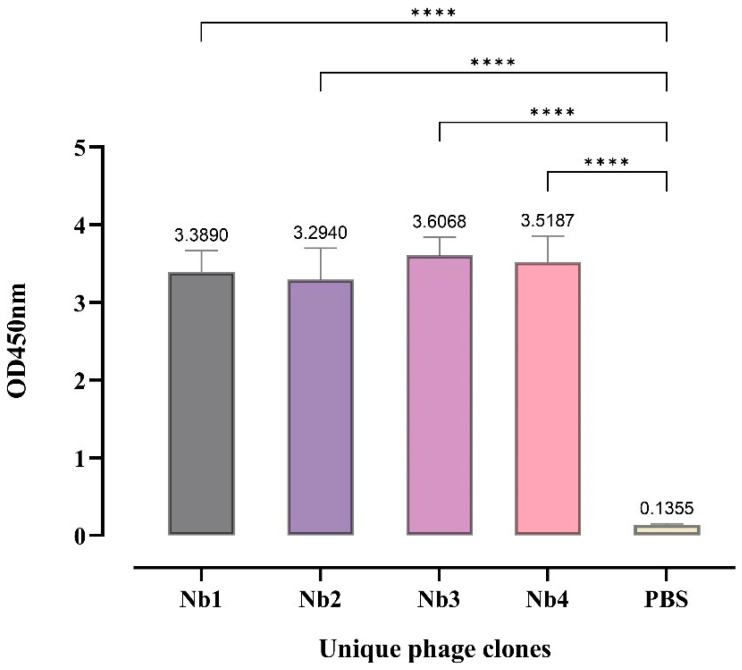
Affinity of four phage clones to the S1 protein. ELISA results showed the affinity of four phage monoclonal clones to the S1 protein, where higher OD450nm values indicate stronger affinity. Data are shown as the mean ± SD of three independent tests. Significant different values were (**** *p* < 0.0001) vs. controls.

**Figure 3:**
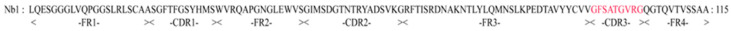
Alignment of amino acid sequences of four nanobodies. The figure highlights the CDR and framework regions, with CDR3 being the binding site of nanobodies to antigens. Therefore, distinguishing between different strains of nanobodies mainly relies on the amino acid sequence of CDR3 (red color). The darker the amino acid color, the higher the conservation.

After infecting TG1 cells with the screened nanobody display vector, 48 monoclonal clones were picked and induced with IPTG for expression. Nanobodies released from the cells were obtained by low-temperature freezing and thawing. Using PEDV S1 protein as the antigen, PBS was added to the negative control wells. The crude nanobody extract was added for incubation, followed by incubation with Rb PAb to E-Tag as the primary antibody and HRP-conjugated secondary antibody. Absorbance at 450 nm was measured after color development (Figure 2). Twenty samples meeting the criteria were selected, and after sequencing and alignment, four nanobodies with strong affinity and high enrichment were obtained (Figure 3).

### 3.4. Expression and Purification of Recombinant Nanobodies

**Figure 4:**
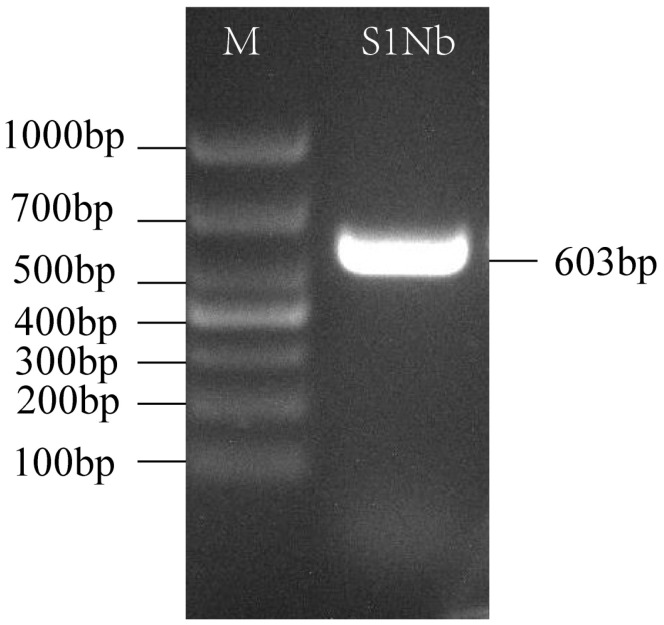
Agarose gel electrophoresis revealed the PCR results for the positive clone. M: DL1000 marker. Lane 1: Positive clone.

**Figure 5:**
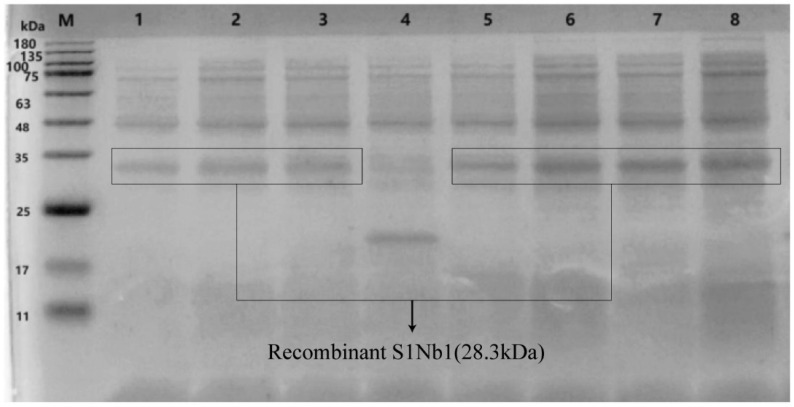
SDS-PAGE revealed the expression of recombinant S1Nb1 in *E. coli* BL21(ED3). M: ColorMixed Protein Marker (11–180 KD). Lanes 1–3: Supernatant from recombinant S1NB1 expressed at 15 °C for durations of 12 h, 15 h, and 18 h, respectively. Lane 4: Expression product of the empty vector. Lanes 5–7: Supernatant of recombinant S1NB1 expressed at 15 °C with IPTG concentrations of 0.1 mM, 0.5 mM, and 1 mM, respectively. Lane 8: Bacterial lysate of recombinant S1NB1.

A nanobody was selected to construct a recombinant expression vector, which had a size of 5079 bp. The size of the resulting fusion protein, SUMO-S1Nb1, was approximately 28.3 kDa. This fusion protein included the TEE translation enhancement element, Strep-Tag II, 6× His-Tag, SUMO solubility enhancement tag, and the nanobody S1Nb1 sequence. The construct was then transformed into *E. coli* BL21 (DE3), and successful transformation was confirmed by PCR analysis (Figure 4). The expression of the recombinant protein under different conditions was analyzed by SDS-PAGE (Figure 5), showing optimal expression at 15 °C with 0.5 mM IPTG induction for 18 h, with soluble protein expression observed in the supernatant.

**Figure 6:**
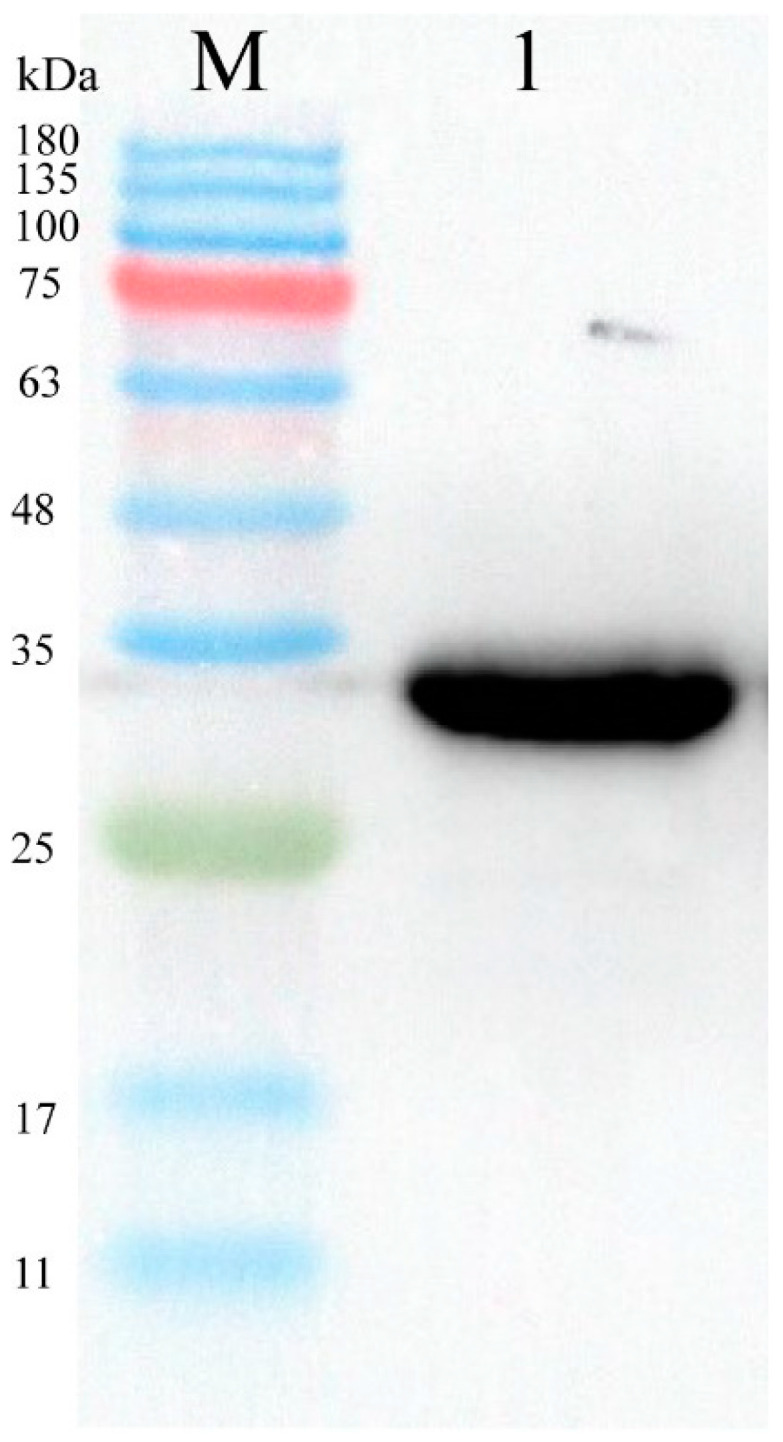
M: ColorMixed Protein Marker (11–180 KD). 1: The Nb has an approximate molecular weight of 28.3 kDa.

His-tagged recombinant proteins were purified using a His-affinity chromatography column. An equilibration buffer containing 10 mM imidazole was used as the column equilibration buffer, and the column was equilibrated with the protein supernatant after mixing in equal volumes. Proteins bound were eluted using an imidazole gradient. Finally, proteins eluted were identified at 80 mM imidazole for eluting impurities and imidazole gradient. Finally, proteins eluted were identified at 80 mM imidazole for eluting impurities and 400 mM imidazole for eluting recombinant proteins and validated using Western blot analysis (Figure 6), Original figures can be found in Supplementary Materials.

### 3.5. Identification of Nanobody

**Figure 7:**
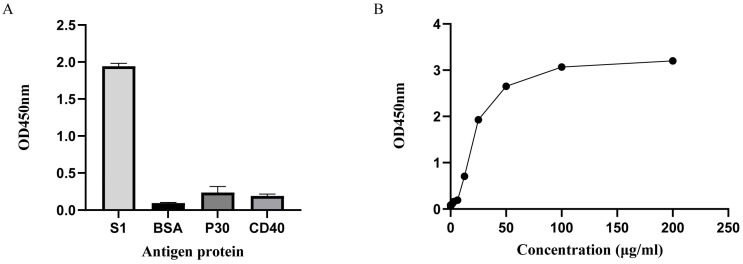
(**A**) shows the specificity of S1Nb1, it has good binding ability with the S1 protein but almost no binding ability with other irrelevant proteins. (**B**) shows the binding ability of S1Nb1, the higher the OD value, the more it binds to the PEDV S1 protein.

The ELISA results indicate that S1Nb1 can bind to the PEDV S1 protein, and there is no cross-reactivity with the BSA, porcine CD40 protein or porcine P30 protein (Figure 7).

## 4. Discussion

Studies have shown that nanobodies can be used for treatment or prevention. Tokuhara et al. [41]. utilized genetically modified rice to express nanobodies against rotavirus, and feeding transgenic rice to mice resulted in excellent preventive effects against rotavirus-induced diarrhea. Nanobodies also have significant advantages in targeted therapy. The use of ADCs is a strategy currently employed in clinical settings for delivering certain chemotherapy drugs [42]. In 2019, caplacizumab [43], developed by Ablynx, a subsidiary of Sanofi, obtained FDA approval for the treatment of acquired thrombotic thrombocytopenic purpura (aTTP), making it the world’s first approved nanobody drug. To date, more ADC drugs have been approved for disease treatment in the United States [44], with over 50 ADC drugs in clinical research stages. These findings indicate the strong specificity of nanobodies. Nanobodies can also be used as detection tools for the rapid and sensitive detection of corresponding antigens. ELISA testing offers advantages such as high sensitivity, ease of operation, and low cost. Lorena Paola Arce et al. [45] selected nanobodies against the HEV-3 ORF2 protein to develop a cELISA method for detecting hepatitis E virus antibodies across multiple species. Nanobodies can also be conjugated with HRP. Zhao et al. [46] utilized this to establish a nanobody-based cELISA detection method for monitoring ASF infection. Wanmei Guo et al. [47] developed a novel optical fiber biosensor using the refractive properties of nanobodies for label-free detection of human epidermal growth factor receptor-2, demonstrating superior characteristics compared to monoclonal antibodies. However, screening for suitable nanobodies is not that simple. Wang et al. expressed a truncated protein of PEDV S1 and screened nanobodies, but the truncated protein of S1 does not include neutralizing epitopes. The screened nanobodies only affect the replication of PEDV. Bao et al. [48]. screened one S protein-specific nanobody (sdAb) S7, but it lacked neutralizing effects. Yang et al. [40] identified a nanobody against the PEDV M protein that can be used as a probe for detection or conjugated with magnetic beads for PEDV isolation.

The pcold vector is an expression vector for low-temperature expression. *E. coli* grows slowly at low temperatures, resulting in slower protein production. This allows sufficient time for proper protein folding, reducing the formation of inclusion bodies. SUMO (small ubiquitin-related modifier) is a powerful fusion tag with a strong solubilization function. SUMO can be fused to the N-terminus of the target protein, greatly enhancing protein stability and solubility. SUMO may promote proper folding of the target protein and reduce its toxicity to cells by acting as a molecular chaperone. SUMO has its highly specific protease (Ulp), which recognizes the tertiary structure of SUMO protein. It cleaves the intact protein after the C-terminal residues of the SUMO tag, releasing it from the fusion protein, and leaving no residual amino acid sequences of the target protein [49,50]. Soluble expression has more advantages than inclusion body expression. The protein is present in the supernatant after bacterial cell lysis, already in a soluble form. We added lysozyme and protease inhibitors to the lysis buffer to facilitate more complete bacterial cell lysis and release more protein. Additionally, protease inhibitors suppress protease activity, reducing protein degradation. After centrifugation, the soluble supernatant can be directly purified avoiding the complex steps of denaturation and refolding. Furthermore, only 22% to 49% of inclusion body proteins regain biological activity after denaturation and refolding. Therefore, nanobodies expressed in soluble form are more likely to possess neutralizing activity [51,52]. However, the purity of soluble supernatant proteins is lower than that of inclusion bodies, and the expression level is relatively low. Only about 2 mg of protein can be purified from 1 g of wet bacterial cells. Using another strain of competent cells such as Rosetta (DE3) may increase expression levels.

Subsequently, we will test whether the nanobody exhibits neutralizing activity. Nanobodies with neutralizing activity will be selected and fused to the pgsA transmembrane protein to construct the pmg36e recombinant vector for expression in Lactococcus lactis. Due to the colonization properties of lactic acid bacteria [53], they can prevent the invasion of porcine epidemic diarrhea virus (PEDV) in the intestinal tract of pigs. Oliver Pusch et al. [54]. expressed cyanovirin-N (CV-N) in Lactobacillus, using Lactobacillus as a mucosal delivery agent to combat HIV-1. Barbara Giomarelli et al. [55]. constructed two recombinant S. gordonii strains secreting or displaying CV-N on the bacterial surface, with CV-N displayed on the bacterial surface efficiently capturing HIV virions. Since PEDV mainly attacks the intestinal system, causing small intestinal mucosal lesions, Lactobacillus colonized in the intestine can form a protective barrier against PEDV.

## 5. Conclusions

In this study, we rescued and screened a nanobody display library stored in our laboratory, identifying four highly specific nanobody sequences. One of these sequences was selected to construct a recombinant expression vector, and a soluble nanobody was expressed and purified. This nanobody exhibited high potency. Future research should emphasize exploring the efficacy of S1Nb1 against PEDV. This nanobody shows great potential as a therapeutic agent for both the treatment and prevention of PEDV. Moreover, S1Nb1 also holds significant promise as a diagnostic tool for the rapid and precise detection of PEDV, facilitating timely interventions to control the disease.

## Figures and Tables

**Table 1:** The titer of eluted phages was determined by observing the number of colonies on the surface of semi-solid culture medium. E/I indicates the proportion of phages displaying a strong affinity to the S1 protein.

Panning Rounds	I	II	III
Input (PFU/mL)	5 × 10^11^	5 × 10^11^	5 × 10^11^
Eluted (PFU/mL)	3 × 10^4^	3.5 × 10^9^	1.24 × 10^10^
E/I	6 × 10^−8^	7 × 10^−3^	2.48 × 10^−2^

## Data Availability

The data that support the findings of this study are available on request from the corresponding author Z.Q. upon reasonable request.

## Supplementary Materials

The following supporting information can be downloaded at: https://www.mdpi.com/article/10.3390/biom14091116/s1, File S1: Western Blotting Figures.

## References

[1] Pensaert M.B., de Bouck P. (1978). A new coronavirus-like particle associated with diarrhea in swine. Arch. Virol..

[2] Pijpers A., van Nieuwstadt A.P., Terpstra C., Verheijden J.H. (1993). Porcine epidemic diarrhoea virus as a cause of persistent diarrhoea in a herd of breeding and finishing pigs. Vet. Rec..

[3] Jung K., Saif L.J., Wang Q. (2020). Porcine epidemic diarrhea virus (PEDV): An update on etiology, transmission, pathogenesis, and prevention and control. Virus Res..

[4] Lin C.M., Saif L.J., Marthaler D., Wang Q. (2016). Evolution, antigenicity and pathogenicity of global porcine epidemic diarrhea virus strains. Virus Res..

[5] Li X., Li Y., Huang J., Yao Y., Zhao W., Zhang Y., Qing J., Ren J., Yan Z., Wang Z. (2022). Isolation and oral immunogenicity assessment of porcine epidemic diarrhea virus NH-TA2020 strain: One of the predominant strains circulating in China from 2017 to 2021. Virol. Sin..

[6] Huang J., Liu H., Qian Y., Wang Z., Tang Q., Cao W. (1980). Study on the transmissible gastroenteritis coronavirus. Agric. Sci. Technol. Shanghai.

[7] Xuan H., Xing D.-k., Wang D.-y., Zhu W., Zhao F., Gong H., Fei S. (1984). Study on the culture of porcine epidemic diarrhea virus adapted to fetal porcine intestine primary cell monolayer. Chin. J. Vet. Sci..

[8] Rosas-Murrieta N.H., Rodriguez-Enriquez A., Herrera-Camacho I., Millan-Perez-Pena L., Santos-Lopez G., Rivera-Benitez J.F. (2024). Comparative Review of the State of the Art in Research on the Porcine Epidemic Diarrhea Virus and SARS-CoV-2, Scope of Knowledge between Coronaviruses. Viruses.

[9] Shen Y., Yang Y., Zhao J., Geng N., Liu K., Zhao Y., Wang F., Liu S., Li N., Meng F. (2022). Molecular epidemiological survey of porcine epidemic diarrhea in some areas of Shandong and genetic evolutionary analysis of S gene. Front. Vet. Sci..

[10] Li C., Li W., Lucio de Esesarte E., Guo H., van den Elzen P., Aarts E., van den Born E., Rottier P.J.M., Bosch B.J. (2017). Cell Attachment Domains of the Porcine Epidemic Diarrhea Virus Spike Protein Are Key Targets of Neutralizing Antibodies. J. Virol..

[11] Sun J., Li Q., Shao C., Ma Y., He H., Jiang S., Zhou Y., Wu Y., Ba S., Shi L. (2018). Isolation and characterization of Chinese porcine epidemic diarrhea virus with novel mutations and deletions in the S gene. Vet. Microbiol..

[12] Bosch B.J., van der Zee R., de Haan C.A., Rottier P.J. (2003). The coronavirus spike protein is a class I virus fusion protein: Structural and functional characterization of the fusion core complex. J. Virol..

[13] Chang S.H., Bae J.L., Kang T.J., Kim J., Chung G.H., Lim C.W., Laude H., Yang M.S., Jang Y.S. (2002). Identification of the epitope region capable of inducing neutralizing antibodies against the porcine epidemic diarrhea virus. Mol. Cells.

[14] Godet M., Grosclaude J., Delmas B., Laude H. (1994). Major receptor-binding and neutralization determinants are located within the same domain of the transmissible gastroenteritis virus (coronavirus) spike protein. J. Virol..

[15] Liu C., Tang J., Ma Y., Liang X., Yang Y., Peng G., Qi Q., Jiang S., Li J., Du L. (2015). Receptor usage and cell entry of porcine epidemic diarrhea coronavirus. J. Virol..

[16] Zhang Y., Chen Y., Zhou J., Wang X., Ma L., Li J., Yang L., Yuan H., Pang D., Ouyang H. (2022). Porcine Epidemic Diarrhea Virus: An Updated Overview of Virus Epidemiology, Virulence Variation Patterns and Virus-Host Interactions. Viruses.

[17] Kong N., Meng Q., Jiao Y., Wu Y., Zuo Y., Wang H., Sun D., Dong S., Zhai H., Tong W. (2020). Identification of a novel B-cell epitope in the spike protein of porcine epidemic diarrhea virus. Virol. J..

[18] Shi W., Hao H., Li M., Niu J., Hu Y., Zhao X., Li Q. (2021). Expression and Purification of a PEDV-Neutralizing Antibody and Its Functional Verification. Viruses.

[19] Shirato K., Maejima M., Islam M.T., Miyazaki A., Kawase M., Matsuyama S., Taguchi F. (2016). Porcine aminopeptidase N is not a cellular receptor of porcine epidemic diarrhea virus, but promotes its infectivity via aminopeptidase activity. J. Gen. Virol..

[20] Li W., Luo R., He Q., van Kuppeveld F.J.M., Rottier P.J.M., Bosch B.J. (2017). Aminopeptidase N is not required for porcine epidemic diarrhea virus cell entry. Virus Res..

[21] Whitworth K.M., Rowland R.R.R., Petrovan V., Sheahan M., Cino-Ozuna A.G., Fang Y., Hesse R., Mileham A., Samuel M.S., Wells K.D. (2019). Resistance to coronavirus infection in amino peptidase N-deficient pigs. Transgenic Res..

[22] Zhang J., Wu Z., Yang H. (2019). Aminopeptidase N Knockout Pigs Are Not Resistant to Porcine Epidemic Diarrhea Virus Infection. Virol. Sin..

[23] Hamers-Casterman C., Atarhouch T., Muyldermans S., Robinson G., Hamers C., Songa E.B., Bendahman N., Hamers R. (1993). Naturally occurring antibodies devoid of light chains. Nature.

[24] Smith G.P. (1985). Filamentous fusion phage: Novel expression vectors that display cloned antigens on the virion surface. Science.

[25] Chakravarty R., Goel S., Cai W. (2014). Nanobody: The “magic bullet” for molecular imaging?. Theranostics.

[26] Muyldermans S. (2021). A guide to: Generation and design of nanobodies. FEBS J..

[27] Hassanzadeh-Ghassabeh G., Devoogdt N., De Pauw P., Vincke C., Muyldermans S. (2013). Nanobodies and their potential applications. Nanomedicine.

[28] Van Heeke G., Allosery K., De Brabandere V., De Smedt T., Detalle L., de Fougerolles A. (2017). Nanobodies(R) as inhaled biotherapeutics for lung diseases. Pharmacol. Ther..

[29] Mitchell L.S., Colwell L.J. (2018). Comparative analysis of nanobody sequence and structure data. Proteins.

[30] Harmsen M.M., De Haard H.J. (2007). Properties, production, and applications of camelid single-domain antibody fragments. Appl. Microbiol. Biotechnol..

[31] Li M., Fan X., Liu J., Hu Y., Huang H. (2015). Selection by phage display of nanobodies directed against hypoxia inducible factor-1alpha (HIF-1alpha). Biotechnol. Appl. Biochem..

[32] Yang E., Liu Q., Huang G., Liu J., Wei W. (2022). Engineering nanobodies for next-generation molecular imaging. Drug Discov. Today.

[33] Dumoulin M., Conrath K., Van Meirhaeghe A., Meersman F., Heremans K., Frenken L.G., Muyldermans S., Wyns L., Matagne A. (2002). Single-domain antibody fragments with high conformational stability. Protein Sci..

[34] Kaur H. (2021). Stability testing in monoclonal antibodies. Crit. Rev. Biotechnol..

[35] Goldman E.R., Liu J.L., Zabetakis D., Anderson G.P. (2017). Enhancing Stability of Camelid and Shark Single Domain Antibodies: An Overview. Front. Immunol..

[36] Peyron I., Kizlik-Masson C., Dubois M.D., Atsou S., Ferriere S., Denis C.V., Lenting P.J., Casari C., Christophe O.D. (2020). Camelid-derived single-chain antibodies in hemostasis: Mechanistic, diagnostic, and therapeutic applications. Res. Pract. Thromb. Haemost..

[37] Salvador J.P., Vilaplana L., Marco M.P. (2019). Nanobody: Outstanding features for diagnostic and therapeutic applications. Anal. Bioanal. Chem..

[38] Bates A., Power C.A. (2019). David vs. Goliath: The Structure, Function, and Clinical Prospects of Antibody Fragments. Antibodies.

[39] Xu Y., Xiong L., Li Y., Xiong Y., Tu Z., Fu J., Chen B. (2015). Anti-idiotypic nanobody as citrinin mimotope from a naive alpaca heavy chain single domain antibody library. Anal. Bioanal. Chem..

[40] Yang S., Li L., Yin S., Shang Y., Khan M.U.Z., He X., Yuan L., Gao X., Liu X., Cai J. (2018). Single-domain antibodies as promising experimental tools in imaging and isolation of porcine epidemic diarrhea virus. Appl. Microbiol. Biotechnol..

[41] Tokuhara D., Álvarez B., Mejima M., Hiroiwa T., Takahashi Y., Kurokawa S., Kuroda M., Oyama M., Kozuka-Hata H., Nochi T. (2013). Rice-based oral antibody fragment prophylaxis and therapy against rotavirus infection. J. Clin. Investig..

[42] Panikar S.S., Banu N., Haramati J., Del Toro-Arreola S., Riera Leal A., Salas P. (2021). Nanobodies as efficient drug-carriers: Progress and trends in chemotherapy. J. Control. Release Off. J. Control. Release Soc..

[43] Morrison C. (2019). Nanobody approval gives domain antibodies a boost. Nat. Rev. Drug Discov..

[44] Schrama D., Reisfeld R.A., Becker J.C. (2006). Antibody targeted drugs as cancer therapeutics. Nat. Rev. Drug Discov..

[45] Arce L.P., Pavan M.F., Bok M., Gutiérrez S.E., Estein S.M., Santos A.T., Condorí W.E., Uhart M.M., Parreño V., Vizoso-Pinto M.G. (2023). A multispecies competitive nanobody-based ELISA for the detection of antibodies against hepatitis E virus. Sci. Rep..

[46] Zhao H., Ren J., Wu S., Guo H., Du Y., Wan B., Ji P., Wu Y., Zhuang G., Zhang A. (2022). HRP-conjugated-nanobody-based cELISA for rapid and sensitive clinical detection of ASFV antibodies. Appl. Microbiol. Biotechnol..

[47] Guo W., Yu Y., Xin C., Jin G. (2024). Comparative study of optical fiber immunosensors based on traditional antibody or nanobody for detecting HER2. Talanta.

[48] Bao F., Wang L., Zhao X., Lu T., Na A.M., Wang X., Cao J., Du Y. (2019). Preparation and characterization of a single-domain antibody specific for the porcine epidemic diarrhea virus spike protein. AMB Express.

[49] Marblestone J.G., Edavettal S.C., Lim Y., Lim P., Zuo X., Butt T.R. (2006). Comparison of SUMO fusion technology with traditional gene fusion systems: Enhanced expression and solubility with SUMO. Protein Sci..

[50] Bird L.E. (2011). High throughput construction and small scale expression screening of multi-tag vectors in *Escherichia coli*. Methods.

[51] Singhvi P., Panda A.K. (2022). Solubilization and Refolding of Inclusion Body Proteins. Methods Mol. Biol..

[52] Singh S.M., Sharma A., Upadhyay A.K., Singh A., Garg L.C., Panda A.K. (2012). Solubilization of inclusion body proteins using n-propanol and its refolding into bioactive form. Protein Expr. Purif..

[53] Berryman M.A., Triplett E.W., Ludvigsson J. (2023). Human leukocyte antigen-dependent colonization of Lactobacillus in the early-life gut. Front. Microbiomes.

[54] Pusch O., Boden D., Hannify S., Lee F., Tucker L.D., Boyd M.R., Wells J.M., Ramratnam B. (2005). Bioengineering lactic acid bacteria to secrete the HIV-1 virucide cyanovirin. J. Acquir. Immune Defic. Syndr..

[55] Giomarelli B., Provvedi R., Meacci F., Maggi T., Medaglini D., Pozzi G., Mori T., McMahon J.B., Gardella R., Boyd M.R. (2002). The microbicide cyanovirin-N expressed on the surface of commensal bacterium Streptococcus gordonii captures HIV-1. AIDS.

